# The Effects of Industrial Protective Gloves and Hand Skin Temperatures on Hand Grip Strength and Discomfort Rating

**DOI:** 10.3390/ijerph14121506

**Published:** 2017-12-04

**Authors:** Mohamed Z. Ramadan

**Affiliations:** 1 Industrial Engineering Department, Faculty of Engineering, King Saud University, P.O. Box 800, Riyadh 11421, Saudi Arabia; mramadan1@ksu.edu.sa; Tel.: +966-11-467-6713; 2 Raytheon Chair for Systems Engineering (RCSE), Advanced Manufacturing Institute, King Saud University, Riyadh 11421, Saudi Arabia

**Keywords:** hand grip strength, muscles, ergonomics, Jamar dynamometer, maximal effort, hand force assessment, subjective rating

## Abstract

Daily working activities and functions require a high contribution of hand and forearm muscles in executing grip force. To study the effects of wearing different gloves on grip strength, under a variety of hand skin temperatures, an assessment of the maximum grip strength was performed with 32 healthy male workers with a mean age (standard deviation) of 30.44 (5.35) years wearing five industrial gloves at three hand skin temperatures. Their ages and anthropometric characteristics including body mass index (BMI), hand length, hand width, hand depth, hand palm, and wrist circumference were measured. The hand was exposed to different bath temperatures (5 °C, 25 °C, and 45 °C) and hand grip strength was measured using a Jamar hydraulic hand dynamometer with and without wearing the gloves (chemical protection glove, rubber insulating glove, anti-vibration impact glove, cotton yarn knitted glove, and RY-WG002 working glove). The data were analyzed using the Shapiro–Wilk test, Pearson correlation coefficient, Tukey test, and analysis of variance (ANOVA) of the within-subject design analysis. The results showed that wearing gloves significantly affected the maximum grip strength. Wearing the RY-WG002 working glove produced a greater reduction on the maximum grip when compared with the bare hand, while low temperatures (5 °C) had a significant influence on grip when compared to medium (25 °C) and high (45 °C) hand skin temperatures. In addition, participants felt more discomfort in both environmental extreme conditions. Furthermore, they reported more discomfort while wearing neoprene, rubber, and RY-WG002 working gloves.

## 1. Introduction

Grip strength is an important aspect of all working activities in daily active life in which workers use their hands in manual operations such as construction material mixing, manual materials handling, carpentry, plumbing and shoveling. In addition, workers who operate hand-held power tools, who are occupationally exposed to hand-transmitted vibration, which is associated with various disorders of the hand and arm [1,2,3], require hand protection using gloves. These operations are likely to occur in outdoor environments. The climatic temperature of some regions around the world varies between 5 °C and 45 °C. Taylor et al. [4], as well as Bedford [5], reported that there is a positive relationship between mean hand skin temperature and the environmental air temperature which influences the body’s thermal exchanges with the thermal environment.

Therefore, ergonomists must consider this environmental issue when designing equipment used by workers in their daily working life due to the fact that hand gripping is an essential element of their activities [6,7]. Gripping is caused by bending all the fingers together except the thumb. Thus, grip strength is defined as the total contact force applied to a handle with his/her maximum voluntary contraction effort. 

Local cooling of the hand decreases manual performance through both physical and neuromuscular pathways [8]. Local cooling decreases flexibility [9] due to increased viscosity within the joints and soft tissues which interferes with smooth joint movements [10]. Cold also affects muscle activity through decreased metabolism utilization, enzyme activity, calcium, and acetylcholine release, and delayed cross-bridge formation [11]. This decreases the contraction velocity and maximal strength [12]. Thus, hand cooling could increase the risk hazards in workplace such as slaughterhouses [13]. Data on healthy adults and hand grip strength, especially with the effect of a hot environment on hand strength, are scarce. A study that assessed hand grip strength in healthy adults from 21 countries found that men in hot countries exhibited intermediate hand grip strength when compared with the highest levels of hand grip strength noted among European and North American populations [14]. Other than this recent study, there is still a dearth of available literature on hand grip strength in hot climates using protective gloves.

Industrial assemblers, machinists, medical doctors, farmers, and construction workers use gloves in different workplaces to protect their arms and hands from chemicals, heat, cold, and physical harm such as cuts and blisters [15,16]. The effect of glove use on hand grip performance has been investigated. Sudhakar and Schoenmarklin [17] concluded that a certain amount of muscle force was lost in the hand–glove interface based on the measurement of normalized peak and mean muscular activities taken from isometric contractions. Fleming et al. [18] indicated that glove type and hand grip contraction had an effect on physiological fatigue and the subjective perception of fatigue. Since it was reported that protective gloves reduce hand heat loss in a cold air environment by 60–90% and those heat losses were 50–100% greater from the fingers than the palm and the back of the hand [19], wearing gloves may be another risk factor among those whose jobs require repetitive motions and large exertion forces on the hand in hot environments. 

Glove use is generally recommended to keep the hands warm and dry and to protect them from many other hazards, provided that this is consistent with safe and effective tool operation. However, users of thicker, stiffer gloves, such as some industrial gloves, could be trading one health risk for another. Knowledge of the effects of gloves on grip strength can help workers, managers, and safety professionals make informed decisions about glove selection and use in the workplace. This knowledge may also lead to improvements in work gloves [20].

Several researchers [21,22,23,24,25,26,27] have reported that grip strength is dependent on many factors. For example, male participants showed greater grip strength than their female counterparts [28,29] due to the difference in body composition such as low muscle mass and high fat mass in females, which leads to decreased grip strength when compared to males. Hand grip strength reduces with advanced age [30,31]. Various researchers [32] also found that grip strength was positively associated with an individual’s nutritional status [33,34]. This finding draws parallel to the findings of anthropometric measurement studies [35]. Furthermore, nutritional status also leads to specific levels of body mass, which in turn has been found to correlate directly to grip strength [36]. Vikram [37] stated that hand grip strength could be predicted by using forearm circumference and hand length for the dominant hand. Fraser [38] and Mohamed [39] stated that there was a significant correlation between grip strength and forearm girth. Incel [40] stated that hand grip strength was higher in right-hand dominant than left-hand dominant groups. However, the work of Reikeras [41] and Roberts [42] reported that there was no significant difference in grip strength of the dominant and non-dominant hand. Research described in References [43,44,45] reported that grip strength was greater in standing than in sitting and the supine posture because of changes in length of the muscle. Su [43] argued that the 180 degree-flexed shoulder had higher grip strength than zero degree flexion. Swanson [44] revealed that the subject’s grip was weaker in the supported arm compared to the unsupported arm. Watson [45] argued that psychological factors such as depression scores were associated with diminished grip strength. Studies by Auyeung et al. [46] and Choudhary et al. [47] found relationships between hand grip strength and mental fitness or cognition. 

Martin [48] stated on variations in the grip strength of the individuals that grip was greater between 6.00 a.m. to 9.00 a.m. and grip strength decreased between 8.00 p.m. and 4.00 a.m. Saud [49] stated that smokers demonstrated reduced grip strength and fast fatigability in comparison to non-smokers. Ruff [50] stated that grip strength started decreasing at 4000 m to 7000 m and abruptly dropped from 7000 m to 9000 m. Deepak [51] found that immersing hands in hot water increased hand grip strength when compared to immersing hands in cold water. However, results of the Barter [52] study stated that there was no correlation between hand grip hold time and a reduction in temperature. Muscular fatigue developed from repetitive hand activities is of particular concern. Burke [53] compared maximum grip strength values to grip strength endurance; the maximum grip strength was approximately twice as much as grip strength endurance. Dianat et al. [54] found a greater thickness of the glove would limit manual hand dexterity, which may discourage the use of gloves by operators, or thereby limit the effective hand grip strength [18]. Generally, workers tackle the reduced hand grip strength by applying higher hand grip force, thus increasing effort. The increased grip effort may increase the risk of hand–arm disorders such as carpal tunnel syndrome [55]. Many studies have investigated the grip strength reduction due to glove use [2,20]. 

Due to the high cost of work injuries, it is important to identify whether wearing gloves in hot environments during grip activities increases the risk of injuries. It is reported by the U.S. Bureau of Labor Statistics [56] that wearing gloves has been proven to reduce the relative risk of injury by 60%. In addition, hand injuries are caused by physical or chemical hazards and result in burns, bruises, abrasions, cuts, punctures, fractures and amputations. Annually, 110,000 lost-time hand injuries are reported. Also, hand injuries force more than one million workers to the emergency room each year. Therefore, determining whether gloves with varied skin hand temperatures affect grip strength is an important step in answering this question and in preventing these injuries. The aim of this study was to determine whether the use of industrial gloves at low and high skin temperatures affects the grip force exertions in a healthy young population when compared with no glove use in the comfort-zone environment.

## 2. Methodology

### 2.1. Participants

Thirty-two healthy male workers participated in the current study from local industries, aged between 23–39 years, with mean and standard deviation (SD) of 30.44 (5.35) years. Workers were eligible to participate after being screened for free hand, upper-limb, or neck injuries. Workers were screened for any neuromuscular disease or injury that might influence their maximum isometric contraction and had no recent or ongoing hand or upper-limb injury. Therefore, only participants without confounding conditions or contraindications were included in the study. They were all right-handed and compensated for participating in this study. The experimental protocol was approved by the University Internal Review Board (E-16-01182). 

The minimum sample size that was required in this study was established by the following formula in ISO 15535 (British Standard, 2003) [57]. The 90% confidence interval was used for the 5th and 95th percentiles since, in most cases, the anthropometric data that are of interest to product development designers are those are between the 5th and 95th percentiles.

(1) $$n\geq {\left(3.006\times \mathrm{CV}/\alpha \right)}^{2}\;\mathrm{and}\;\mathrm{CV}=S\times 100/\bar{x}$$

In this formula, *n*, CV, $\bar{x}$, S, α represents the sample size, coefficient of variation, the sample mean and standard deviation, and percentage of the desired relative accuracy, respectively. The sample size was computed based on the assumption that a relative accuracy of 10% was sufficient for the 5th and 95th percentiles, and used the empirical means and standard deviations (434 N and 40.3 N with CV = 9.285) from the results of the initial pilot study of 10 participants. The minimum required sample size was determined as 32.

### 2.2. Measurement and Instrumentation

#### 2.2.1. Anthropometric Measurements

Body mass (kg) was measured using a balance scale wearing light clothing, without any footwear. Height (m) was measured with each worker in an upright position in front of a wall looking ahead and heels touching one another. Body mass index (BMI) was computed using the formula: $\mathrm{BMI}=\mathrm{mass}\left(\mathrm{kg}\right)/\mathrm{height}{\left(m\right)}^{2}.$ The forearm circumference of each participant was measured using a flexible fiberglass tape. The hand length was measured from the mid-point of the distal wrist crease to the tip of the middle finger with the hand held straight and stiff. Hand width was measured as the maximum breadth across the palm of the hand (at the distal ends of the metacarpal bones) [58]. Palm length was measured as the length of a straight line from the folded line at the level of the lateral spot on the wrist to that near the middle finger. Mid-upper arm circumference was the circumference of the upper arm measured at the mid-point between the tip of the elbow and the tip of the shoulder, at the olecranon process and acromion. A Siber Hegner GPM Anthropological Instrument was used in this experiment. This instrument consisted of the following: (1) fixed anthropometry (0–2100 mm with straight probes and curved measuring branches); (2) sliding caliper (Martin type length 0–200 mm with depth of 0–50 mm); (3) spreading caliper with rounded ends (0–600 mm); (4) fiberglass tape (Dean, 0–1500 mm); and (5) balance scale (Seca 708, 0.1–200 ± 0.1 kg). 

After receiving an explanation of the study goals and procedures, all candidate workers filled a personal data sheet and brief medical history form to ensure that they were healthy enough to participate in the study. The anthropometric measurements of the participants were collected. The hand lengths (perpendicular distance from a line drawn between the styloid processes to the tip of the middle finger using a sliding caliper), the hand widths (projected distance between radial and ulnar metacarpals at the level of the metacarpal heads from the second to the fifth metacarpal using a sliding caliper), and the wrist circumferences (circumference of wrist at the level of the styloid processes of the radius and ulna, with the hand, outstretched using a tape measure) were measured. In measuring these dimensions, ISO 7250-1, 2008 [59] was followed. Anthropometric measurements of the participants are summarized in Table 1.

#### 2.2.2. Grip Strength

**Figure 1:**
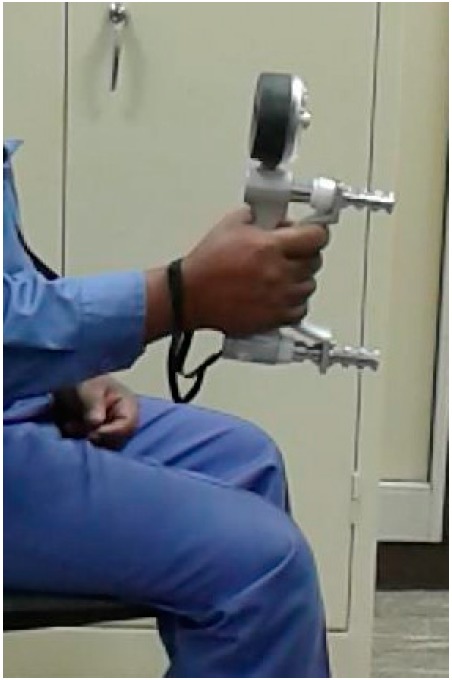
Measurement of the grip hand force using the JAMAR dynamometer with the second handle position (span = 45 mm).

Hand grip strength (HS) has been widely used as a functional parameter of the upper limbs (UL) and general health. The measurement of HS by dynamometry is a low-cost, non-invasive method of simple applicability, widely used in pulmonary rehabilitation and critical care units. Grip strength is caused by bending all the fingers together except the thumb, and is one of the standard practices in evaluating hand function. This paper chose maximum grip strength, which measures the force exerted in Newton, as the maximum voluntary contraction (N) sustained for at least three seconds, and which was often based on a hand-handle interface with a hand grip [60,61]. The Jamar hand grip dynamometer (Model 5030J1, Sammons Preston Rolyan, Bolingbrook, IL, USA) with an adjustable handle span of five grip positions from 35–86 mm (1.375–3.375 inches) in 12.7 mm (0.5 inch) increments, was used by each participant so that the participant’s fingertip forces were completely applied to the dynamometer handle [62] (Figure 1). A single dynamometer was used for all measurements. This dynamometer was chosen as it has been reported to be the most accurate for measuring grip strength [63,64] and standardized test protocols have been developed for its use [63]. A new Jamar dynamometer was purchased specifically for use in this study and unused prior to it. The dynamometer was initially calibrated by the manufacturers, and field calibration was checked for a needle-starting point on zero before each testing session.

Each participant was given verbal instruction and a demonstration before being tested and additional encouragement was provided during the test to exert the maximal grip in the dominant hand by squeezing his fingers and thumb together as hard as possible. The dominant hand was determined by which hand the participant used the most in activities of daily living. The participants were tested in a sitting position, with arms straight out and inclined downward, as shown in Figure 1. Each participant was then asked to hold the hand grip dynamometer tightly for approximately three seconds and then relax. This measurement was performed three times [65], with an interval of one minute between each measurement, and the highest score (N) was considered as the participant’s grip strength score. After consecutively performing this operation, the participant was allowed to rest for at least five minutes before the next treatment.

#### 2.2.3. Glove Types

**Figure 2:**
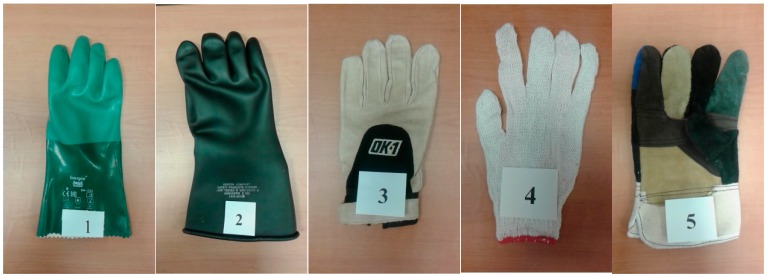
Industrial gloves used in the study. Chemical protection glove (**1**); Rubber insulating glove for electrical shock (**2**); Anti-vibration impact glove (**3**); Cotton yarn knitted glove (**4**); RY-WG002 working glove (**5**).

A chemical protection glove (Scorpio^®^ 08-352, Neoprene dipped coating on an interlock knit liner); a rubber insulating glove for protection of workers from electrical shock; an anti-vibration impact glove; a cotton yarn knitted glove (30% polyester plus cotton, 70% white blended yarn) for cold anti-freeze, hot, and anti-oil protection; and an RY-WG002 working glove (cow split palm, green double palm, stripe cotton back, rubberized cuff, 10.5"), were used in this study as shown in Figure 2. These gloves are commonly used in industries, construction sites, chemical, and mechanical facilities [20]. These different gloves were selected to represent a broad use in industry and may indicate that one glove type may have different applications for occupational and safety engineers whose workers use gloves in the workplace. The properties of these gloves are shown in Table 2. The workers were allowed to choose the size of gloves that best fit their hands. All gloves were new and unused prior to the study. The gloves differed in the isolation material coating, which included different types of covering materials. These accounted for differences in overall thickness of the gloves. The thickness of the industrial gloves in the palm and finger regions was measured under different applied loads, which are summarized in Table 2.

#### 2.2.4. Hand Skin Temperature and Time Control

**Figure 3:**
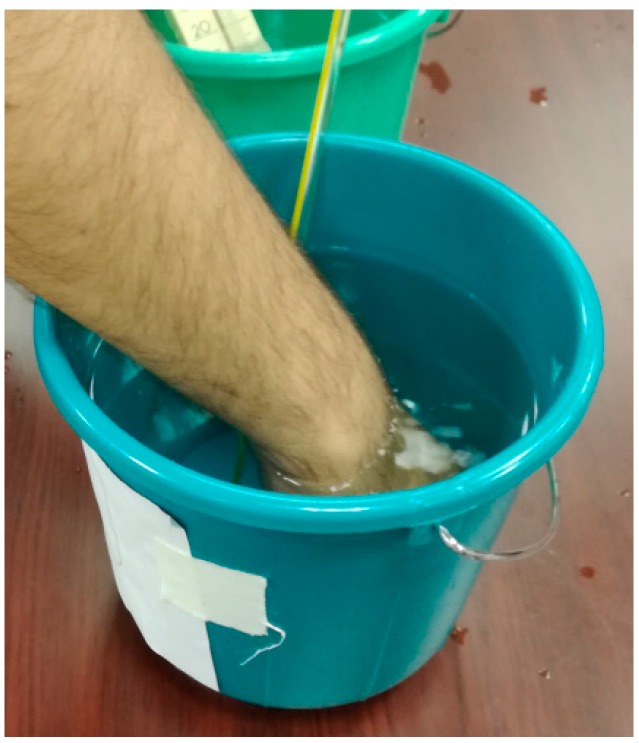
Submerging in cold water bowl (5 °C).

Three thermometers were used to control the temperature degrees of water contained in three bowls. The water at three temperature levels was 5 °C, 25 °C, and 45 °C as shown in Figure 3. A stopwatch was used to schedule the rest time. 

#### 2.2.5. Discomfort Rating

The discomfort ratings were based on locally perceived discomforts (LPDs) in the hands. Groenesteijn et al. [66] proposed a discomfort rating system consisting of three 12 cm-long lines corresponding to pain, numbness and pressure, and tiredness in the relevant body part. The collected data sheet contained those three lines for the full hand–wrist region. The participant was asked to express his discomfort on each of these three measures by marking the corresponding line at the start of each trial and immediately after executing the task. These ratings were quantified on a 20-point scale ranging from zero (no discomfort) to 20 (extreme discomfort). Discomfort ratings are easy to use and require almost no training [67]. The increase in discomfort rating was considered for the statistical analysis.

### 2.3. Experimental Design

A within-subject design was used in this study where hand grip strength was the dependent variable and the independent variables were glove type and hand skin temperature, in order to determine their effects on grip strength in the six-day study. The model used was analysis of variance (ANOVA), and bare hand grip strength at the beginning of each testing day was measured. The glove type was set to six (bare hand and wearing five gloves); and three temperature-controlled water baths (maintained at 5 °C, 25 °C, and 45 °C) were used to alter the participants’ hand skin temperatures. If the trial was dealing with gloves, the participant tried on the most suitable glove size before performing the trial. Three treatments were tested on separate days. The order of testing was randomly assigned. After establishing the normality of the data using the Shapiro–Wilk parametric test, regression analysis with the Pearson correlation coefficient was used to determine the influence of the anthropometric data on hand grip strength. All statistical analyses were performed using SPSS software (version 22) (IBM, Armonk, NY, USA), while a significant difference among the data was considered (*p* < 0.05). The Tukey test was used to differentiate and find the significant differences between the factor levels. ANOVA was also used to identify the significance of the different glove and hand skin temperature treatments on the maximum grip strength values and exertion rating.

### 2.4. Procedures

To make participants familiar with the equipment, protocol, and procedures for collecting grip strength data, a simplified show was presented on how to use the dynamometer properly and adjust the handle of the dynamometer as appropriate with the participant’s hand. The trials started according to the sequence as planned in a randomized block design, with every participant considered as a block. 

Before the actual testing sessions, the participants performed several trial tests with self-determined submaximal grip forces at the five different grip span settings of the dynamometer to determine their “preferred hand grip span” where they felt comfortable to produce their maximal grip strength. Haidar et al. [68] recommended asking the participant after the initial determination of the preferred hand grip span to perform hand grip strength more than once in order to try the spans one above and one below the preferred span so as to ensure that it was the one the participant actually preferred. This method of providing one more and one less, and the opportunity for the participant to decide on “preferred span” was an improvement over the previously used method of subjective determination of preferred span. Eksioglu [69] used both objective and subjective methods of determining the preferred span with no significant difference.

During the session, the test started by asking the participant to submerge his hand in a temperature controlled water bath for two minutes when switching from one treatment to another and needed to increase or decrease the hand skin temperature by 20 °C, or four minutes when increasing or decreasing to 40 °C where finger skin temperature changed quite rapidly. A study by Morton and Provins [70] reported that the finger temperature of their participants dropped to −5 °C in a period of 3–4 min (at a rate of around 10 °C/min) [71]. Hence, the short exposure of two minutes that we started with may have been sufficient to reduce the finger/hand temperature to the modeled muscle temperature by 20 °C [71]. Hand skin temperature was not measured in this study.

However, previous researchers [72,73] have suggested that the hand skin temperature can be assumed to be close to the water bath temperature [74]. After the participant dried his wet hand using soft tissues, he was asked to wear the assigned glove. Next, the worker was seated in a chair free from armrests, and upright against the back of a chair with a smooth horizontal floor. The shoulder was adducted and neutrally rotated, the elbow flexed at 90 degrees, forearm in neutral, and the wrist at 15 degrees to 30 degrees of extension [75]. This protocol was recommended by the American Society of Hand Therapists (ASHT; ISO 10819, 2013) [76]. The Jamar dynamometer was inserted into the worker’s hand and the test procedure explained. Workers were then asked to grasp using the inner hand surface and fingers on the handle at the Jamar dynamometer. All workers were instructed to provide maximal effort for each grip. From the start until the end of the trial, the participant exerted maximum effort with his dominant hand against the dynamometer handle. The obtained reading from the dynamometer display was recorded and then reset to the initial point. The participant was asked to redo the trial under the same conditions, with two more trials for each condition [77]. Participants were given one-minute rest breaks between each trial. Finally, the participant had a rest break of five-minutes between testing treatments. To reduce the effects of muscle fatigue, measurements were limited to a maximum of three hand treatments a day. Furthermore, each participant was asked to perform a bare hand grip strength test at the beginning of each day to ensure that no effect of the hand muscle being trained throughout the experimental execution sessions. The test session was canceled and rescheduled in the event that the participant reported fatigue.

Grip strength testing was performed on all workers with a bare hand and while they wore gloves separately. The sequence of the grip strength testing with and without gloves was randomly assigned. The investigator trained and instructed workers in performing the grip tests, and administered all examinations. The ergonomics laboratory climate was kept at normal room temperature (~22 °C) and about normal humidity (~50%) for all participants. Due to their potential effects on the participants’ motivation and performance, competition, noise, spectators, etc. were avoided. As time of day has been found by some researchers to affect grip strength, testing was carried out from 10:00 a.m. and 5:00 p.m. This was done to avoid early morning testing, which has been found to produce abnormal readings [78].

## 3. Results

One-way ANOVA was carried out to test whether the effect of executing the experiment daily could increase hand muscle strength, which may influence the experimental results. It was found that no significant differences existed among the testing days, as shown in Table 3. This allowed us to analyze the data without considering the covariant factor.

### 3.1. Hand Skin Temperature

**Figure 4:**
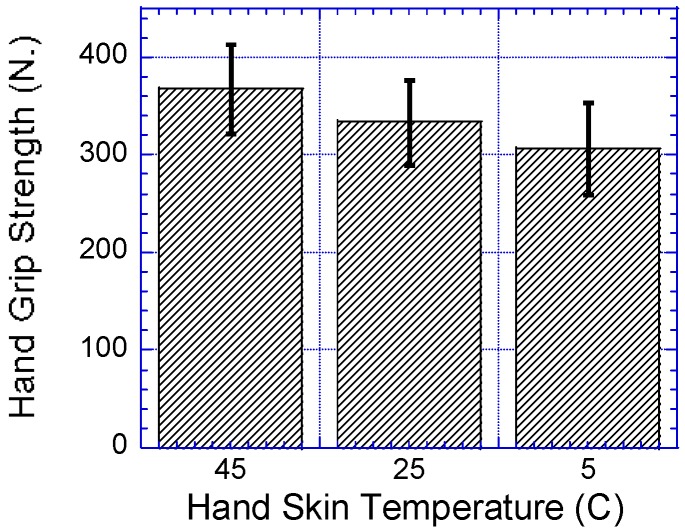
Effect of hand skin temperatures on HS.

**Figure 5:**
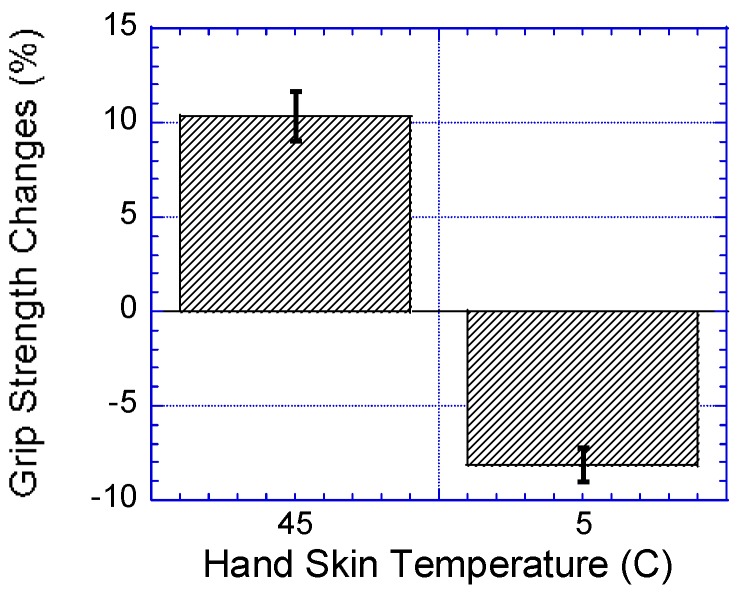
Comparison of grip strength percentage reduction due to cooling skin temperature, and percentage increase in grip strength due to raising hand skin temperature when compared to 25 °C hand skin temperature. Bar errors represent the standard deviations.

As shown in Table 4, the results showed that hand skin temperature had a significant effect on the participants’ HS, F (2,62) = 22.413, *p* < 0.0001, Γ^2^ = 0.42. The participants’ HS decreased significantly from high skin temperatures to low. All pair-wise comparisons were significant at *p* < 0.0001 as shown in Figure 4 and Figure 5.

### 3.2. Glove Type

**Figure 6:**
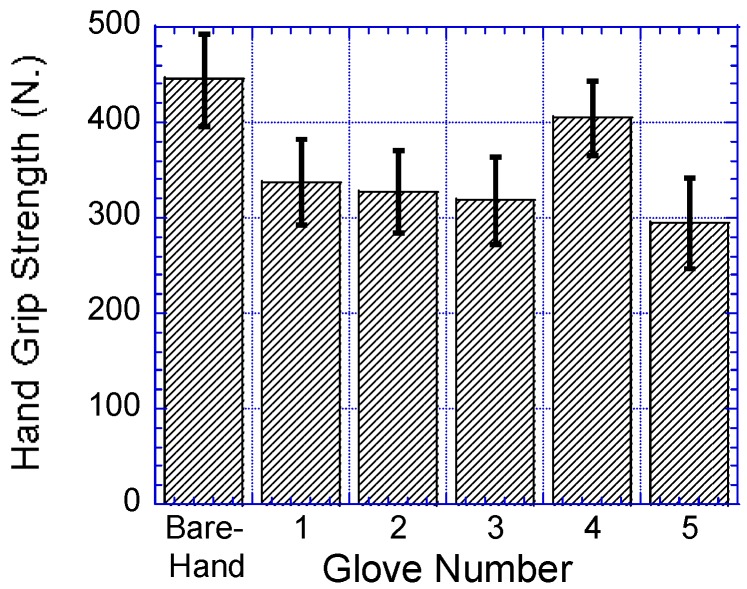
Mean grip strength for the five gloves plus bare-hand used in the study. Bar errors represent the standard deviations.

**Figure 7:**
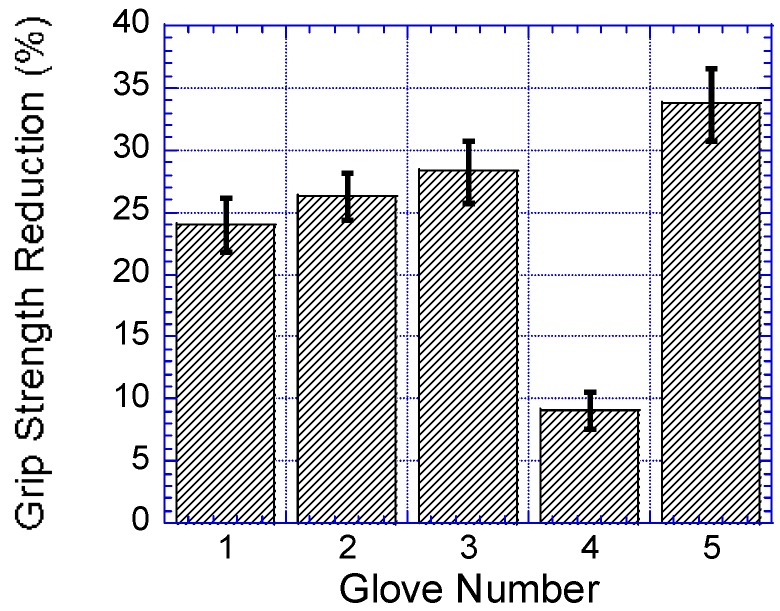
Comparison of mean grip strength percentage reduction of the five gloves when compared to the bare-hand grip strength. Bar errors represent the standard deviations.

As shown in Table 5, wearing gloves had a significant effect on participants’ hand grip strength, F (5155) = 148.527, *p* < 0.0001, Γ^2^ = 0.827. The participants’ hand grip strengths were significantly reduced when the participants wore gloves when compared to their grip strengths bare-handed, as shown in Figure 6. As shown in Table 4, results of the post hoc comparisons showed that no significant differences in grip strengths when the participants wore rubber insulating and anti-vibration impact gloves, as well as cotton yarn knitted and anti-vibration impact gloves. As shown in Figure 7, the comparison of mean grip strength percentage reduction of the five gloves was compared to the bare-hand grip strength.

A regression analysis was carried out to figure out the relationship between glove thickness and the hand grip strength. The result showed that a good correlation existed between glove thickness and hand grip strength with a Pearson correlation of −0.724, *p* < 0.0001. The regression analysis revealed the following equation: Grip strength (N) = 433.95 – 40.28 × glove thickness (mm).

### 3.3. Anthropometrics and Grip Strength

The relationship between the anthropometric data and grip strength in terms of Pearson correlation is shown in Table 6. Weight, BMI, and forearm circumference showed a highly significant positive correlation with grip strength in the dominant hand (*p* < 0.0001). In addition, height was significantly correlated with grip strength (*p* < 0.001). On the other hand, there was no significant correlation between age, hand length, palm length, and hand depth with grip strength (*p* > 0.05).

### 3.4. Discomfort Rating

**Figure 8:**
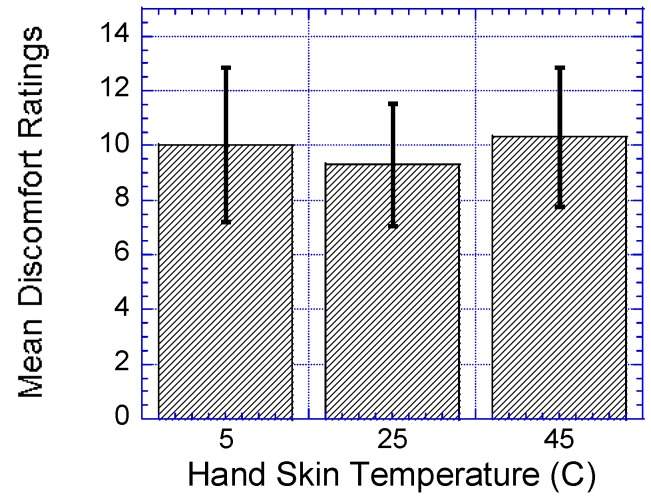
Effect of hand skin temperatures on discomfort ratings. Bar errors represent the standard deviations.

As shown in Table 7, results showed that the hand skin temperature had a significant effect on the participants’ discomfort rating, F (2,62) = 9.603, *p* < 0.0001, Γ^2^ = 0.237. The participants’ discomfort ratings increased significantly at hand skin temperatures of 5 °C and 45 °C when compared to hand skin temperature of 25 °C, *p* < 0.002 and *p* < 0.001, respectively, as shown in Figure 8.

Furthermore, as shown in Table 7, wearing gloves had a significant effect on the participants’ discomfort rating, F (5155) = 6.276, *p* < 0.0001, Γ^2^ = 0.168. The participants felt significant discomfort when they wore glove numbers 1, 2, and 5 when compared to their feeling bare-handed and wearing glove numbers 3 and 5 based on pair-wise comparisons (Table 8).

## 4. Discussion

This study investigated the effects of local cooling and heating of the hand on hand grip strength using five different industrial gloves. The results showed that hand cooling had a greater influence on decreasing hand grip strength. This result agreed with the result obtained by Cheng et al. [9], which showed a reduction in hand strength after cooling to 14 °C. However, this study disagreed with the Cornwall [79] result where they found that little or no change in muscle strength occurred when the muscle temperature changed from 27 °C to 40 °C. Furthermore, it was found that no interaction existed between glove type and hand skin temperature on grip strength.

Another point that should be considered during product design is where lowering hand temperature could cause the worker to generate a force output greater than necessary due to the loss of cutaneous sensation. This unpredictable force generation could increase the risk of musculoskeletal disorders when hand grip strength is lower; therefore, it is necessary to warm the hands and maintain a reasonable tactile sensitivity to avoid less hand force generated in hand strength or lowering the demands of hand strength. Alternatively, if working in a cold environment is not avoidable, increasing the duration of pauses could be an alternative method to alleviate hand muscular fatigue.

It is known that thick gloves limit manual hand dexterity, which may discourage workers to use gloves [80]. In addition, gloves, in general, require an increase in grip effort, and thereby limit the effective hand grip strength. Usually, operators tackle reduced hand grip strength by applying higher hand grip force and thus increasing the muscular effort. The increased grip effort may increase the risk of hand–arm disorders such as carpal tunnel syndrome. This study found a good correlation between glove thickness and hand grip strength. It showed comparable grip strength reduction for all gloves (11.3–50.1%). The greatest reduction was obtained with the RY-WG002 working glove. This result of decreasing hand grip strength with increasing glove thickness was consistent with the results from previous studies [3,20].

Based on the results of this study, it was concluded that personal anthropometric variables such as forearm circumference, weight, height and BMI had significant correlations with hand grip strength. The correlation between anthropometric data and hand grip strength obtained in this study coincided with the findings of previous studies [23,32,81]. However, in other studies [82,83,84], no or weak correlations were found between the anthropometric data and grip strength. It is believed that the findings of the present study will be useful for product designers to design and develop ergonomic products that will cater to the needs of workers.

## 5. Conclusions

Hand grip strength is essential in manual operations and the activities of daily life, but the influence of hand skin temperature and wearing a protective glove on hand grip strength has not been well documented. Therefore, this study investigated the effects of five typical industrial gloves on grip strength under three different environmental conditions. The five types of gloves used in different industrial settings such as chemical, electrical, vibrated parts, mechanical and construction units were investigated in this study. The experiment confirmed that glove use generally reduces the grip strength applied to Jamar handles. In addition, glove thickness was identified as one of the main factors that influenced grip strength. This study found that strength reductions due to high glove thickness were more severe than those of thinner gloves, regardless of hand skin temperature. This suggests that the replacement of some types of regular work gloves used at some workplaces with appropriately selected ones may not greatly increase the grip effort required to operate a machine or a tool; however, the benefits should be carefully evaluated before replacing work gloves with thinner gloves. Knowledge of the effects of gloves and hand skin temperature on grip strength can allow designers, ergonomists, and safety professionals to make correct decisions regarding glove selection and use in the workplace, especially in extreme environments. This may also lead ergonomists to work harder to improve glove characteristics.

### Limitations

Some limitations of the present study should be highlighted. First, the gender and age of the participants, who were younger than most of the working population, limited the generalization of the results to the population [85]. This may help designers to obtain a better understanding of gender differences in underlying mechanisms. The mechanism of blood flow and cross-sectional areas of the thenar and hypothenar muscles could be included in such types of study, and may further add a new dimension when exploring the highest benefit.

## Figures and Tables

**Table 1:** Depicts the participant information (*n* = 32).

Measurement	Mean	SD	Min–Max
BMI (kg/m^2^)	23.06	2.58	17.89–28.22
Body mass (kg)	69.74	9.19	50.0–83.8
Height (cm)	173.78	5.83	163.6–186.4
Hand length (cm)	19.92	1.3	17.9–21.9
Hand width (cm)	8.73	0.69	7.5–9.8
Forearm circumference (cm)	17.69	1.55	15.6–21.0
Palm length (cm)	10.33	0.52	9.5–11.2
Hand depth (cm)	3.3	0.36	2.7–3.8

**Table 2:** Properties of the used industrial glove.

Glove	Thickness at Palm (mm)	Thickness at Fingers (mm)
Chemical protection glove #1	2.8	2.7
Rubber insulating glove for electrical shock #2	2.6	1.9
Anti-vibration impact glove #3	3.0	2.6
Cotton yarn knitted glove #4	1.2	1.2
RY-WG002 working glove #5	3.3	3.6

**Table 3:** Post hoc comparisons for measuring hand grip strength daily before collecting data. *

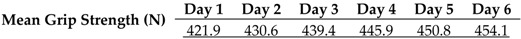

* Underlined days denote the means not significantly different at α = 0.05.

**Table 4:** Results of the analysis of variance (ANOVA) for grip strength measured at three hand skin temperatures and six glove conditions (bare-hand and five glove conditions).

Source	SS	DF	MS	*F*	*p*	Partial Eta
						Squared
HST	840.222	2	420.111	22.413	0.000	0.420
Error	1162.111	62	18.744			
Glove	14,961.467	5	92.293	148.527	0.000	0.827
Error	3122.700	155	20.146			
HST × Glove	182.694	10	18.269	0.881	0.551	0.028
Error	426.972	310	20.732			

**Table 5:** Post hoc comparisons for glove type plus bare-hand. *

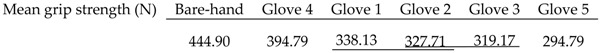

* Underlined gloves denotes the mean was not significantly different at α = 0.05.

**Table 6:** Correlation between anthropometric data and grip strength.

Anthropometrics Measure	Pearson Correlation	*p* Value
Age	0.160	0.382
Weight	0.956	0.0001
Height	0.541	0.001
BMI	0.808	0.0001
Hand length	0.072	0.695
Hand width	0.375	0.035
Forearm circumference	0.892	0.0001
Palm length	0.150	0.412
Hand depth	0.164	0.369

**Table 7:** Result of the ANOVA for discomfort rating measured at three hand skin temperatures and the six glove conditions (bare-hand and five glove conditions).

Source	SS	DF	MS	*F*	*p*	Partial Eta
						Squared
HST	104.431	2	52.215	9.603	0.000	0.237
Error	337.125	62	5.438			
Glove	180.743	5	36.149	6.276	0.000	0.168
Error	892.813	155	5.760			
HST × Glove	59.215	10	5.922	0.881	0.806	0.025
Error	2277.229	310	7.346			

**Table 8:** Post hoc comparisons for glove type plus bare-hand. *

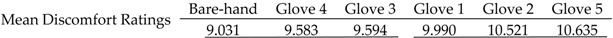

* Underlined gloves denote the means not significantly different at α = 0.05.
